# Prolonged stress alters the PC1/PC2 ratio in the rat lateral hypothalamus, implicating impaired orexin maturation

**DOI:** 10.22038/IJBMS.2024.76858.16620

**Published:** 2024

**Authors:** Mohadeseh Salami, Mahmoud Elahdadi Salmani, Taghi Lashkarbolouki

**Affiliations:** 1 School of Biology, Damghan University, Damghan, Iran

**Keywords:** Hypothalamic-pituitary-adrenal system, Orexins, Prohormone convertase, Rat, Stress

## Abstract

**Objective(s):**

Stress elicits physiological and neuroendocrine responses mediated by the hypothalamic-pituitary-adrenal (HPA) axis and lateral hypothalamus (LH). However, prolonged stress can dysregulate neuropeptide systems like orexin. This study investigated the effects of temporary and prolonged stress on HPA activity and orexin processing in the rat LH.

**Materials and Methods:**

Male Wistar rats were exposed to various stress repetitions. The stress paradigm is defined as short (acute; 1 day and mild; 3 days) and long (sub-chronic; 10 days and chronic; 21 days)-term 6 hr daily restraint stress. Plasma corticosterone (CORT) served as an index of HPA function. Expression of prepro-orexin and its processing enzymes prohormone convertases (PC) 1 and 2 was measured in LH tissues using semiquantitative RT-PCR.

**Results:**

The plasma level of CORT was elevated following mild, sub-chronic, and chronic, but not acute stress versus unstressed controls. The expression of prepro-orexin was heightened following all stress exposures. However, PC1 increased and PC2 decreased only after prolonged stress. The PC1/PC2 ratio was also selectively augmented with sub-chronic and chronic stress, implying impaired orexin maturation.

**Conclusion:**

Together, these data demonstrate that the HPA axis and lateral hypothalamic orexin system respond to stress based on stress repetition. Changes in orexin processing enzyme mRNA, exclusively after chronic stress, imply potential effects on peptide maturation, requiring confirmation of the orexin production at the protein level.

## Introduction

Stress arises from threats to homeostasis and elicits adaptive physiological and behavioral responses (1). However, chronic stress can have damaging effects on physical and mental health, including increased risk for obesity, cardiovascular disease, depression, and other disorders (2). In our previous study, we observed an inconsistency in the effects of acute and chronic stress on the lateral hypothalamus (LH) area of the brain. Acute stress caused anxiety behaviors, while chronic stress led to a decrease in the number of neurons in the LH region (3). At the molecular level, stress exposure causes widespread changes in gene expression across multiple brain regions (4). Understanding these molecular alterations provides critical insight into how prolonged stress impairs brain function and contributes to stress-related pathologies.

The hypothalamus is a brain region that plays a key role in regulating the stress response through the hypothalamic-pituitary-adrenal (HPA) axis and sympathetic nervous system activation (5). In particular, LH contains orexin/hypocretin neurons that promote arousal and alertness in response to stressors (6, 7). Orexin peptides act in widespread brain regions to modulate stress reactivity, energy balance, reward processing, and other functions (8). Chronic stress has been associated with dysregulation of the orexin system, including altered orexin levels in cerebrospinal fluid (9, 10) and impaired reactivity of orexin neurons in the LH area (11). Animal studies also show that chronic stress impairs spatial learning and memory, which correlates with decreased orexin expression in the hippocampus (12, 13). In humans, orexin deficiency causes the sleep disorder narcolepsy, which is characterized by excessive daytime sleepiness, cataplexy, and cognitive impairments (14). Rodent models with reduced orexin signaling exhibit deficits in depression- and anxiety-like behaviors (15, 16). This further indicates a key role for orexin in regulating appropriate emotional and cognitive responses. Together, these findings indicate that prolonged stress disrupts normal orexin signaling, which may contribute to the adverse effects of stress on cognition and brain health. Despite strong evidence that chronic stress dysregulates the orexin system, the precise molecular mechanisms underlying these alterations remain unclear. Because of the importance of LH in modulating stress reactivity and associated sequelae, elucidating mechanisms underlying stress effects on orexin signaling could provide new insights into anxiety, depression, addictive disorders, cognitive decline, and sleep disturbances associated with chronic stress. 

The process of normal orexin production begins with the transcription of the prepro-orexin gene to RNA, which is then translated into a 131 amino acid prepro-orexin protein. This protein is subsequently cleaved into two transcripts, orexin-A (OXA; 33 amino acids) and orexin-B (OXB; 28 amino acids)(17), by two peptidases, prohormone convertase (PC) 1 and 2 (18). PCs are enzymes that cleave precursor proteins into their mature, active forms through proteolytic processing (19). This includes the maturation of neuropeptide precursors, making PCs crucial regulators of neuropeptide production and secretion (20). PCs are 20 times more concentrated in the orexin neurons of the LH area and it is known that OXA is cleaved by PC1 and OXB is cleaved using PC2 (21-23). However, little is currently known about the effects of stress on PCs and their role in regulating orexin peptide expression. Given the putative function of orexin in promoting hyperarousal during stress, examining the impact of stress on orexin synthesis and maturation could uncover novel mechanisms underlying stress-induced orexin dysregulation.

In this study, we will investigate the effects of escalating acute, mild, sub-chronic, and chronic stress paradigms on HPA activity, expression of prepro-orexin, and its processing enzyme PC in the rat LH area. Furthermore, this study will also calculate the ratio of PC1 to PC2 expression, which examines the net effect of stress on the overall capacity for orexin maturation, rather than just viewing the enzymes in isolation. This integrative approach allowed us to evaluate the combined influence of stress on both processing enzymes, rather than just the individual changes in each one. Finally, illuminating these molecular changes could provide new insights into how stress disrupts orexin signaling, while also identifying potential intervention targets for mitigating adverse neurobehavioral effects of chronic stress. This work will expand our fundamental understanding of how repeated stress alters LH gene regulation of orexin production to impact brain functions connected to orexin physiology. 

## Materials and Methods

***Animals***

The present study used male Wistar rats weighing between 200 and 250 grams as subjects. The rats were obtained from the Razi Institute in Karaj, Iran. All experimental procedures complied with the guidelines of the National Institute of Health Guide for the Care and Use of Laboratory Animals (1996) and the ethical standards for animal research of Damghan University. The rats, four per cage, were housed under standard laboratory conditions consisting of a 12-hour light/12-hour dark cycle with lights on at 7 am. They had free access to food and water.

***Stress paradigm***

The rats were restrained in a separate and specially designated room, not in their home cages. This ensured a controlled environment for the stressor application, distinct from their regular living space. The control rats, which did not experience stress, were not in close proximity to other rats undergoing restraint. They were kept in a separate area away from the stressed group to ensure they were not influenced by the stressor. 

The stress protocol involved restraining rats in Plexiglas tubes for 6 hr daily starting at 10 am. The stress conditions were divided into short-term or temporary (acute and mild) and long-term or prolonged (sub-chronic and chronic) groups. The acute stress model consisted of a single day of restraint stress. The mild stress group experienced restraint for 3 consecutive days. The sub-chronic group underwent 10 days of repeated restraint stress. Finally, the chronic stress model involved 21 days of daily restraint sessions. This range of stress exposure periods allowed comparison of both temporary and prolonged stress effects.

***Experimental groups***

Animals were divided into 5 experimental groups (n=4 rats per group for PCR measurements, n=6 rats per group for CORT). The groups consisted of:

• Control (Ctrl): Naive rats living in the same cage as other groups.

• Acute stress (S1): Rats exposed to one session of restraint stress and sampled the next day.

• Mild stress (S3): Rats were exposed to three restraint sessions over three consecutive days and sampled on the fourth day.

• Sub-chronic stress (S10): Rats exposed to ten restraint sessions over ten days and sampled on day 11.

• Chronic stress (S21): Rats exposed to twenty-one restraint sessions over 21 days and sampled on day 22.

Blood plasma was collected for corticosterone (CORT) measurements. Hypothalamus tissue was collected from each rat for analysis of PC1, PC2, and prepro-orexin mRNA expression. The hypothalamus was precisely dissected under a stereomicroscope to isolate specific regions containing orexin neurons, based on established neuroanatomical maps (24). To extract the lateral and posterior zones known to be enriched in orexin-containing neurons, the hypothalamus was bisected midsagittally after removal. The right hemisphere was further segmented by making a diagonal cut from the dorsal medial region to the ventral lateral side (25). All dissection was carried out on ice with sterile instruments to preserve tissue integrity. Extracted sections were immediately frozen at -70 ^°^C until further processing.

***Plasma CORT measurement***

After conducting the experiments, the animals were deeply anesthetized using ketamine (90 mg/kg, IP) and Xylazine (6.0 mg/kg, IP). There were six animals per group. To prevent any potential effects of anesthesia on the measured variables, the animals were quickly decapitated. Trunk blood (2 ml) was collected into a syringe containing 0.25 ml of sodium citrate anticoagulant. The collected blood samples were then centrifuged at 4000×g for 15 min at 4 ^°^C. The resulting supernatant plasma was stored at -70 ^°^C until further analysis.

To assess plasma CORT levels, a fluorescence-based assay (Spectrofluorometer Jasco 6200, Japan) was performed (26, 27). Initially, CORT standard solutions were prepared in concentrations ranging from 400 to 0.1 ng/ml (Supplementary figure). To extract the CORT content from the plasma samples, a mixture of chloroform and methanol (2:1 v/v) was added to the samples. The mixture was then combined with 3 ml of chloroform, vortexed, and centrifuged at 2000×g. The bottom layer was mixed with 300 µl of 0.1 N NaOH, vortexed, and centrifuged again at 2000×g, to extract CORT into an aqueous phase. The resulting bottom layer was added to a mixture of 3 ml of a solution containing sulphuric acid and ethanol in a 4:1 ratio, followed by centrifugation at 2000×g, to convert CORT into a fluorometric derivative. The bottom layer was incubated in darkness for 5 min and then measured fluorometrically using fixed excitation and emission wavelengths of 472 nm and 525 nm, respectively, based on prior optimization studies. The emission at 525 nm was recorded during excitation at 472 nm. The CORT concentration in the samples was determined by comparison to a standard curve generated with known CORT concentrations between 0.1 - 400 ng/ml.

***Gene expression measurement using semiquantitative RT-PCR***

In our study, we quantified the mRNA levels of the common precursor, prepro-orexin, which is responsible for the generation of both OXA and OXB peptides. The PCs, PC1 and PC2, are enzymes responsible for cleaving prepro-orexin to form OXA and OXB. To design the PCR primers, we referred to previously published studies (28, 29) and used BLAST to ensure the specificity of the primers and to avoid non-specific amplification. LH tissue samples were lysed in the lysis buffer of RNA extraction using the RNX plus kit -acts based on phenol-chloroform isolation- from SinaClon (IRAN) per the manufacturer’s protocol. Extracted RNA was run on a 0.75% agarose gel. RNA concentration was measured upon its absorbance in 260 nm and its quality and integrity upon a ratio of 260/280 nm and 0.5% agarose gel electrophoresis. One μg of total RNA was used in the first strand cDNA synthesis kit of SinaClon according to instructions. The cDNA was amplified by PCR using the following cycling conditions: initial denaturation at 94 ^°^C for 15 sec, annealing at 57 ^°^C for PC1 and PC2, 64 ^°^C for prepro-orexin, and 55 ^°^C for GAPDH for 1 min each, extension at 72 ^°^C for 2.5 min for 30 cycles total using an Eppendorf Mastercycler semiquantitative PCR system. PCR primers against prepro-orexin (442 bp product), PC1 (206 bp product), PC2 (226 bp product), and the housekeeping gene GAPDH (131 bp product) were used to specifically amplify cDNA targets. A 100 bp ladder was used to confirm product sizes. The specific PCR primer sequences were: prepro-orexin forward 5’-CCTTCCTTCTACAAAGGTTCCC-3’, reverse 5’- TGGTTACCGTTGGCCTGAA-3’; PC1 forward 5’-CTGTTGGCTGAAAGGGAAAG-3’, reverse 5’-TGCTTCATGTGTTCTGGCTG-3’; PC2 forward 5’-TTGGCTACGGAGTCCTTGAT-3’, reverse 5’-CTGGTTGC GTTGACTGTGAT-3’; GAPDH forward 5’-TGACATCAAGAAGGTGGTGAAGC -3’, reverse 5’-CCCTGTTGCTGTAGCCGTATTC-3’. The PCR products were analyzed by 1.5% agarose gel electrophoresis and band densitometry was performed using image J software. Target gene expression was normalized to the internal control; GAPDH.

***Data analysis***

ANOVA was used to compare averages between multiple independent groups, which was followed by *post hoc* comparisons using the Tukey test when the ANOVA was statistically significant. All the statistical tests were performed using GraphPad Prism software V. 9.3.0. The level of signiﬁcance was *P*<0.05 and the data are presented as mean±SEM.

## Results

This study evaluated the effect of different degrees of restraining stress on plasma CORT levels, and the expression of prepro-orexin, PC1, and PC2 in the LH brain tissue of rats.

***Repeating the stress sessions augmented the plasma CORT***

**Figure 1:**
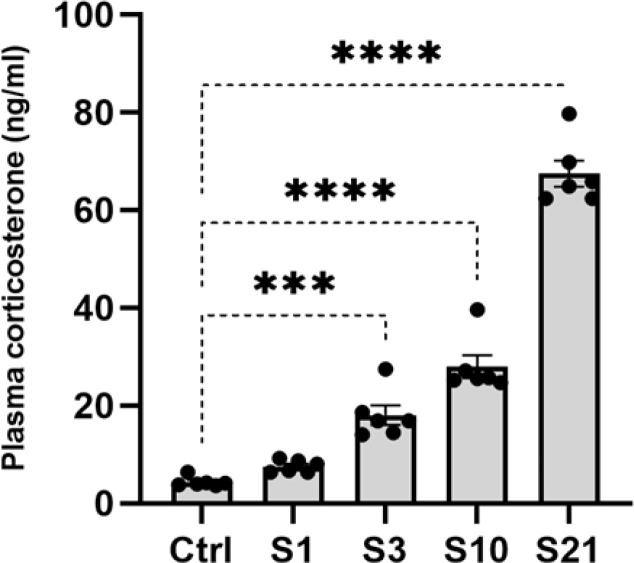
Plasma Corticosterone (CORT) levels following exposure to acute, mild, sub-chronic, and chronic stress to rats

Plasma CORT levels were compared between five groups-naive control animals and those subjected to acute, mild, sub-chronic, or chronic stress-using one-way ANOVA. This analysis revealed a significant difference in CORT levels across groups (F4, 25=188.24, *P*<0.0001). *Post* *hoc *Tukey tests showed that mild, sub-chronic, and chronic stress significantly increased plasma CORT compared to naive controls (*P*<0.001). However, acute stress did not lead to elevated CORT versus unstressed animals. These results indicate that repeated sessions of stress exposure are required to augment circulating CORT, while a single session of (acute) stress did not affect this hormone plasma level (Figure 1). Overall, the plasma CORT data demonstrates an increasing stress response dependent on the repetition of restraint stressors.

***Stress increased the expression of prepro-orexin in the lateral hypothalamus***

**Figure 2:**
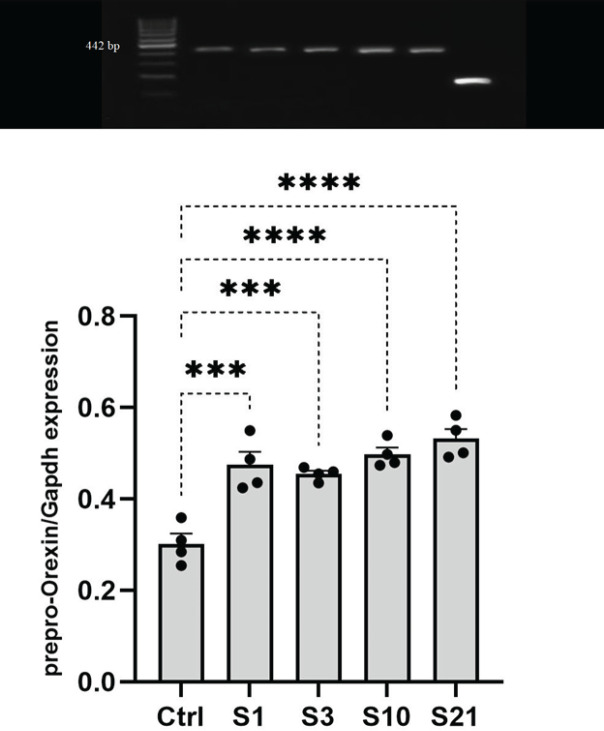
Prepro-orexin expression in the lateral hypothalamus following stress exposure to rats

To evaluate the effect of stress on prepro-orexin expression in the LH, RT-PCR was performed on RNA extracted from this brain region. One-way ANOVA demonstrated a significant increase in prepro-orexin expression in stressed animals compared to unstressed controls (F4, 15=19.21, *P*<0.001). *Post hoc* Tukey tests revealed that acute, mild, sub-chronic, and chronic stress groups all showed heightened prepro-orexin expression versus controls (*P*<0.001). These findings indicate that stress exposure, regardless of duration or repetition, up-regulates prepro-orexin transcription in the LH (Figure 2). The consistent increase across acute to chronic stress groups suggests prepro-orexin induction may represent an early and sustained hypothalamic response to restrain stress. Overall, this data provides evidence that stress activates orexin-producing neurons in the LH, which may have downstream effects on arousal, wakefulness, and stress physiology.

***Stress increased the expression of PC1 in the lateral hypothalamus***

**Figure 3:**
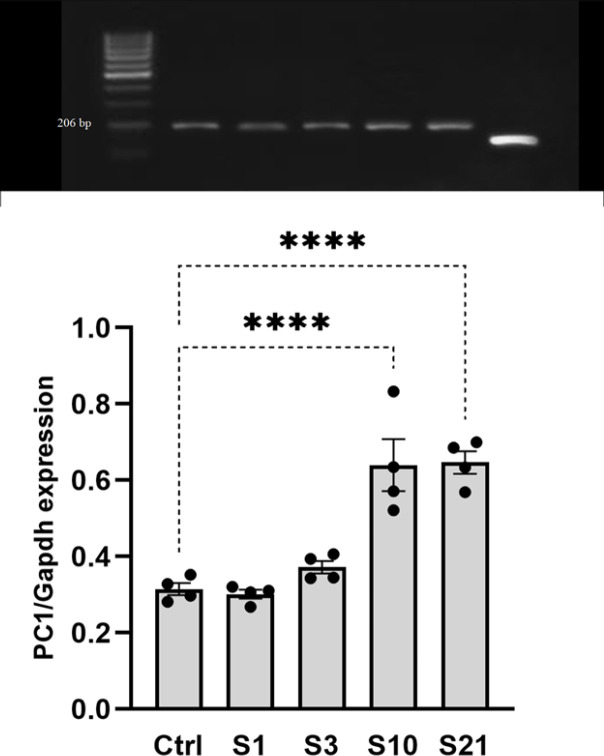
PC1 expression in the lateral hypothalamus following rat exposure to stress

PC1 gene expression in the lateral hypothalamic tissue was assessed using RT-PCR following RNA extraction. A one-way ANOVA test revealed a significant difference in PC1 expression between the groups (F4, 15=24.42, *P*<0.001). A *post hoc* Tukey test showed that PC1 expression was significantly higher in the sub-chronic and chronic stress groups compared to the control, acute stress, and mild stress groups (*P*<0.001). Acute or mild stress did not significantly alter PC1 expression in the lateral hypothalamic tissue compared to controls, as seen in Figure 3. In summary, long-term stress induced a substantial up-regulation of PC1 expression, whereas short-term stress had no notable effect (Figure 3).

***Stress decreased the expression of PC2 in the lateral hypothalamus***

**Figure 4:**
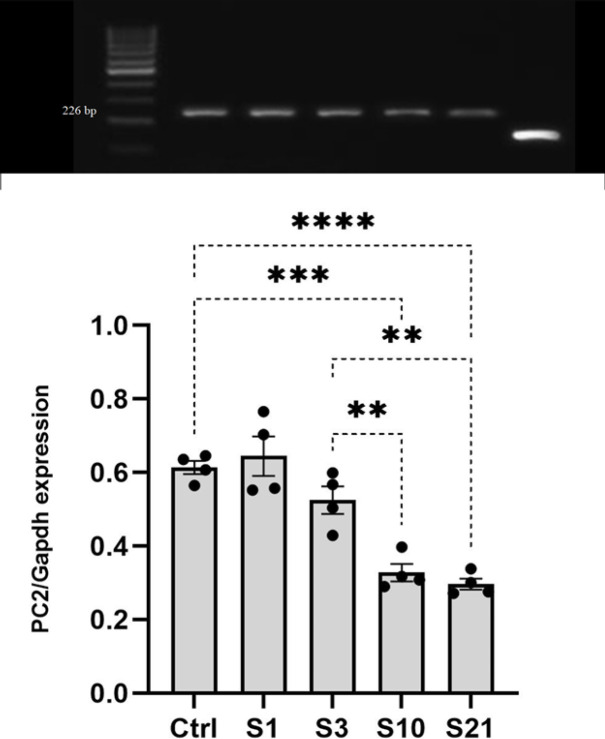
Differential PC2 gene expression levels in the lateral hypothalamus under different stress conditions of rats

RT-PCR analysis of lateral hypothalamic tissue following RNA extraction revealed that PC2 gene expression was significantly different between groups based on a one-way ANOVA test (F4, 15= 23.97, *P*<0.001). A *post hoc* Tukey test further showed that PC2 expression levels were markedly lower in the sub-chronic and chronic stress groups compared to the control group (*P*<0.001 for both), the acute stress group (*P*<0.001), and the mild stress group (*P*<0.01). Acute or mild stress did not visibly alter PC2 expression in the lateral hypothalamic tissue relative to controls, as seen in Figure 4. In summary, long-term stress was associated with a notable decrease in PC2 expression whereas short-term stress had no apparent effect (Figure 4).

***Stress increased the ratio of PC1/PC2 in the lateral hypothalamus***

**Figure 5:**
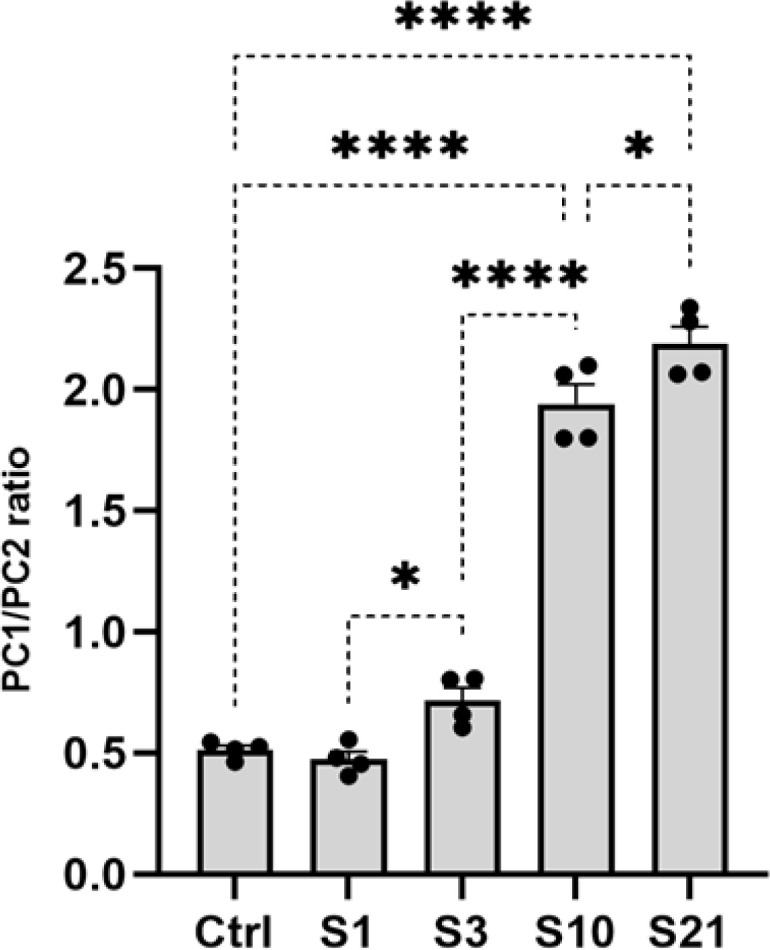
Comparison of PC1/PC2 expression levels in the rat lateral hypothalamic tissue among stress groups

By examining the ratio of PC1 to PC2 expression, we could assess the net effect of stress on the overall capacity for orexin maturation. Reverse Transcriptase-PCR analysis of lateral hypothalamic tissue revealed a significant difference in PC1/PC2 expression between groups according to a one-way ANOVA test (F4,15=222, *P*<0.001). A subsequent Tukey *post hoc* test showed that PC1/PC2 expression increased from mild stress to sub-chronic stress and chronic stress animals compared to acute stress (*P*<0.05), mild stress (*P*<0.001), and sub-chronic stressed (*P*<0.05) animals, respectively. Only sub-chronic and chronic stress significantly increased the PC1/PC2 ratio compared to control animals (*P*<0.001). As shown in Figure 5, stress intensity correlated positively with changes in the PC1/PC2 expression ratio, with the most pronounced effects seen after sub-chronic and chronic stress (Figure 5).

In summary, temporary stress had no effect on PC1/PC2 expression, whereas sub-chronic and chronic stress markedly increased the PC1/PC2 ratio in LH.

## Discussion

The present study aimed to evaluate the effect of acute, mild, sub-chronic, and chronic stress on HPA axis activity and prepro-orexin processing in LH.

Plasma CORT, a marker of HPA axis activity, showed an increasing response based on stress repetition. It increased following mild, sub-chronic, and chronic stress but not acute stress. Prepro-orexin expression, a marker of orexin neuron activation, increased after all stress exposures: acute, mild, sub-chronic, and chronic stress. This suggests acute stress is sufficient to activate orexin neurons and this activation is sustained with stress repetitions. The effects on expression of the orexin processing enzymes, PC1 and PC2 differed based on stress condition. PC1 expression increased, while PC2 expression decreased after prolonged (sub-chronic and chronic) stress. This demonstrates the differential effects of stress conditions on these enzymes that may modulate orexin peptide maturation. Notably, the ratio of PC1 to PC2 increased after prolonged (sub-chronic and chronic) stress conditions. The changing ratio of these processing enzymes over time suggests that stress may alter the production of active orexin neuropeptides in prolonged stress conditions.

***Repeated, but not acute, stress exposure increased CORT***

Stress elicits physiological responses mediated by interconnected systems in the central nervous system and periphery (30). The HPA axis plays a key role through glucocorticoid release, like CORT in rodents (30, 31), which serves as a biomarker of stress response (31). This study examined plasma CORT following temporary (acute or mild) and prolonged (sub-chronic and chronic) stress in rodents. Results showed increased CORT after mild, sub-chronic, and chronic but not acute stress compared to unstressed controls. This suggests repeated stress exposure over time augments circulating CORT, while single acute stress does not (Figure 1). These data agree with previous findings of increased CORT following chronic stress (32). However, the lack of CORT elevation after acute stress contrasts with our previous works after electric shock to animals (10) and indicates the stress response may depend on the type of stress in addition to its repetition. Importantly, our single stress session likely does not fully capture the peak CORT levels and recovery kinetics following acute versus chronic stress. Future studies should characterize the dynamic hormonal response at earlier post-stress time points. Further study on how stress exposure patterns influence HPA activity and outcomes is warranted.

***Stress increased prepro-orexin expression in the LH***

Orexin neurons regulate arousal, wakefulness, stress response, and other vital functions (33). Examining stress effects on prepro-orexin expression in the LH provides insights into stress-induced changes in orexin signaling (34). This study found increased prepro-orexin expression in the LH after temporary (acute or mild) and prolonged (sub-chronic and chronic) stress. These data agree with previous studies demonstrating stress-induced up-regulation of orexin (35-37). The consistent prepro-orexin increase across stress groups suggests early and sustained activation of orexin-producing neurons during stress (38). While another work found blunted prepro-orexin response after chronic stress (39), this study indicates an increase regardless of stress repetition. Together, these findings support that stress, regardless of repetition, activates LH orexin neurons (37, 38, 40), which may impact all targets of orexin neuron’s structure and function (35). Elucidating mechanisms underlying stress effects on orexin signaling could provide insights into anxiety, depression, and sleep disturbances (3). However, additional research is needed to understand the mechanisms by which stress affects orexin levels and their resulting functional consequences.

***Chronic, but not acute, stress increased PC1 expression in the LH***

The PC gene expression leads to PC enzymes that process prepro-orexin into mature orexin peptides (18, 41). Examining stress effects on PC1 expression in the LH provides insights into stress-induced changes in orexin signaling. This study found increased PC1 expression in the LH after prolonged (sub-chronic and chronic), but not temporary (acute or mild) stress. These data agree with previous work demonstrating stress-induced PC1 up-regulation (30). The selective PC1 increase after prolonged stress suggests a discriminative hypothalamic response to chronic stress. While acute stress increased prepro-orexin expression (10, 37, 38), the lack of simultaneous change in PC1 indicates mature orexin production may not be altered through this mechanism or there might be alternative mechanisms involved in orexin production. On the other hand, PC1 up-regulation with chronic stress could suggest that it takes time for the orexin processing mechanisms to be altered by stress. However, further study is needed to determine if increased PC1 translates to greater orexin levels, as orexin production was not measured in this study. In a study by Nilaweera *et al*. on obese animals, there was a decrease in PC1 expression in the obese animals compared to lean ones, while PC1 expression recovered due to leptin infusion in the obese animals. Colocalization of PC1 and prepro-orexin expression in the LH neurons demonstrate a functional link between PC1 and orexin production (21). Additional research should investigate mechanisms underlying chronic stress effects on orexin signaling and maturation.

***Chronic, but not acute, stress decreased PC2 expression in the LH***

The PC2 gene is involved in processing prepro-orexin into mature orexin peptides (18). Examining stress effects on PC2 expression in the LH provides insights into stress-induced changes in orexin signaling. This study found decreased PC2 expression in the LH after prolonged (sub-chronic and chronic), but not temporary (acute or mild), stress exposure. While acute stress increased prepro-orexin transcription (10, 37, 38), PC2 levels were unchanged, suggesting mature orexin production may not be altered by short-term stress. In contrast, the down-regulation of PC2 with prolonged stress indicates orexin signaling could be disrupted by chronic stress exposure. These data contrast with the up-regulation of PC1 after chronic stress, highlighting differential effects on orexin processing enzymes. While we observed increased CORT levels and altered prepro-orexin processing with chronic stress, the connection between these two outcomes remains unclear. An important limitation of the current study is that we did not directly investigate the functional relationship between elevated CORT (and HPA axis activation) with changes in orexin processing enzymes. While we observed parallel increases in CORT and altered PC1 and PC2 expression with chronic stress, the causal link between these factors remains speculative. CORT may directly regulate the expression of orexin processing genes, or HPA overactivation may affect the corticotropin-releasing hormone (CRH)-receptor 1 through the action of CRH (3). The reductions in PC2 specifically point to possible deficits in mature orexin peptide levels that may impact anxiety, depression, and sleep disorders seen with chronic stress (3, 42). Future studies should explore whether manipulated CORT levels modulate PCs and orexin maturation. Pharmacological blockade of CORT signaling specifically in the hypothalamus could also elucidate whether preventing HPA axis activation mitigates effects on the orexin system. Establishing a clear mechanistic relationship between CORT and orexin processing deficits would strengthen the significance of our findings by linking HPA axis dysfunction to impaired neuropeptide regulation. Understanding the precise role of elevated CORT in disrupting orexin processing may reveal novel intervention targets to mitigate the adverse effects of chronic stress.

***Prolonged (sub-chronic and chronic) stress increased the PC1/PC2 ratio in the LH***

Assessing the ratio of PC1 to PC2 expression provides a means to evaluate the collective impact of stress on both processing enzymes. This study evaluated PC1 and PC2 expression in the LH following temporary (acute, mild) and prolonged (sub-chronic and chronic) stress exposure. Results showed increased PC1/PC2 ratio after prolonged (sub-chronic and chronic) but not temporary (acute, mild) stress. Stress repetition correlated positively with PC1/PC2 ratio changes, with the most pronounced effects after prolonged stress (Figure 5). 

While acute stress increased prepro-orexin transcription (10, 37, 38), the lack of PC1/PC2 change suggests mature orexin levels may not be impacted. The stress response triggers various mechanisms and circuits, with CORT being just one of them and not the sole factor responsible for stress-induced changes in orexin production. We believe that temporary stress may not have the capacity to alter the orexin processing pathway and the involved PCs. Instead, temporary stress may influence the release of vesicles, thereby increasing the release of orexins. In contrast, the increased PC1/PC2 ratio with chronic conditions indicates possible augmentation of orexin signaling and inequality of OXA and OXB production (23) that may influence anxiety, depression, and sleep disorders (3, 42). Therefore, the impact of stress on orexin production and processing is not solely attributed to CORT, and different stress repetitions may engage distinct mechanisms in regulating orexin production and release. 

Given the heterogeneity of the hypothalamus and the presence of other neuropeptides, the functional impact of the mRNA changes on orexin processing requires further investigation. While our study provides valuable insights into the impact of stress on orexin processing enzymes, it is important to confirm these changes at the protein level. To further validate our findings, we need to examine the orexin protein level and PC enzymatic activity as the functional level in future directions. This will allow us to determine whether the observed alterations in mRNA expression translate to changes in protein levels and activity. By doing so, we can gain a more comprehensive understanding of the impact of stress on orexin processing and signaling.

Compared to naive controls, mild (*P<*0.001), sub-chronic and chronic stress (*P<*0.0001) significantly increased plasma CORT levels. Acute stress did not alter CORT levels relative to unstressed animals. ***P<0.001 and ****P<0.0001 in comparison to the Ctrl

Ctrl: Control; S1: Acute stress; S3: Mild stress; S10: Sub-chronic stress; S21: Chronic stress

Acute and mild (*P<*0.001), and sub-chronic and chronic (*P<*0.0001) stress significantly increased prepro-orexin mRNA levels compared to unstressed controls. These data demonstrate that diverse stressors uniformly enhance prepro-orexin transcription. The first lane is the ladder, the 2nd to 6th is prepro-orexin, and the right-most bar in the jell picture represents the GAPDH. ***P<0.001 and ****P<0.0001 in comparison to the Ctrl

Ctrl: Control; S1: Acute stress; S3: Mild stress; S10: Sub-chronic stress; S21:Chronic stress

Sub-chronic and chronic stress significantly increased PC1 mRNA levels compared to control, acute stress, and mild stress groups (*P<*0.0001). Acute and mild stress did not alter PC1 expression relative to unstressed controls. The first lane is the ladder, the 2nd to 6th is PC1, and the right-most bar in the jell picture represents the GAPDH. ****P<0.0001 in comparison to the Ctrl

Ctrl: Control; S1: Acute stress; S3: Mild stress; S10: Sub-chronic stress; S21: Chronic stress; PC1: Prohormone convertase 1

The graph demonstrates that acute or mild stress does not visibly alter PC2 expression compared to the control group. However, sub-chronic (*P<*0.001) and chronic (*P<*0.0001) stress groups exhibit significantly lower PC2 expression levels compared to the control group. This suggests that long-term stress exposure leads to a notable decrease in PC2 expression in the lateral hypothalamus. The first lane is the ladder, the 2nd to 6th is PC2, and the right-most bar in the jell picture represents GAPDH. ****P<0.0001, ***P<0.001 and **P<0.01 in comparison to the Ctrl.

Ctrl: Control; S1: Acute stress; S3: Mild stress; S10: Sub-chronic stress; S21: Chronic stress; PC2: Prohormone convertase 2

The RT-PCR analysis revealed a significant increase in PC1/PC2 expression in sub-chronic and chronic stress (*P<*0.0001) compared to control animals. There was an increase of chronic to sub-chronic (*P<*0.05), sub-chronic to mild (*P<*0.0001), and mild to acute (P<0.05) stress groups. ****P<0.0001 and *P<0.05 in comparison to the Ctrl

Ctrl: Control; S1: Acute stress; S3: Mild stress; S10: Sub-chronic stress; S21: Chronic stress; PC1: Prohormone convertase 1; PC2: Prohormone convertase 2

## Conclusion

This study illustrates that both HPA axis activity (CORT increase) and orexin processing in the LH (prepro-orexin expression profile) are specifically altered by stress in nearly all stress repetitions. However, the increase in PC1 and decrease in PC2 (resulting in a PC1/PC2 increase) exclusively in chronic conditions suggest a complex regulation of orexin production by stress. This implies that acute stress conditions may affect the production pathway through another stress-activated mechanism (s) without the involvement of CORT, and high CORT levels in chronic conditions may affect the PC1/PC2 in a concentration-dependent manner. A strength of this study is the comparison of multiple stress patterns; however, direct orexin peptide measurements are lacking. In the future, we will focus on examining the enzymatic activity and protein synthesis of PCs, along with the protein synthesis of orexin A and orexin B. Subsequent studies should also investigate the functional consequences of stress-induced changes in orexin signaling further.

## Authors’ Contributions

M S conducted the project, contributed to the execution of the experiments, played a significant role in the data collection and analysis process. M ES principal investigator, designed the project, supervised the research, and was responsible for the overall planning and direction of the study. T L expert advisor, contributed to the development and execution of the RT-PCR and CRT measurements, and provided valuable guidance and expertise throughout the project.

## Conflicts of Interest

The authors declare that they have no conflicts of interest associated with this study.
